# Epidemiological and Molecular Investigation of Ocular Fungal Infection in Equine from Egypt

**DOI:** 10.3390/vetsci7030130

**Published:** 2020-09-08

**Authors:** Amin Tahoun, Helmy K. Elnafarawy, Ehab Kotb Elmahallawy, Abdelhamed Abdelhady, Amira M. Rizk, Hanem El-Sharkawy, Mohamed A. Youssef, Sabry El-Khodery, Hussam M. M. Ibrahim

**Affiliations:** 1 Department of Animal Medicine, Faculty of Veterinary Medicine, Kafrelshkh University, Kafrelsheikh 33511, Egypt; amin12_veta@yahoo.com; 2 Department of Internal Medicine and Infectious Diseases, Faculty of Veterinary Medicine, Mansoura University, Mansoura 35516, Egypt; helmykamal@mans.edu.eg (H.K.E.); mohamed.youssef@mans.edu.eg (M.A.Y.); khodery@mans.edu.eg (S.E.-K.); 3 Department of Zoonoses, Faculty of Veterinary Medicine, Sohag University, Sohag 82524, Egypt; 4 Department of Biomedical Sciences, University of Leon, s/n, 24071 León, Spain; 5 Parasitology and Animal Diseases Department, National Research center, Dokki, Giza, 12622, Egypt; afanrc@yahoo.com; 6 Department of Bacteriology, Mycology and Immunology, Faculty of Veterinary Medicine, Benha University, Benha 13518, Egypt; dr_az80@yahoo.com; 7 Department of Poultry and Rabbit Diseases, Faculty of Veterinary Medicine, Kafrelsheikh University, Kafrelsheikh 33511, Egypt; hanem_amin@yahoo.com

**Keywords:** epidemiology, molecular, characterization, keratomycosis, eye, equine

## Abstract

Diagnosis and treatment of ocular fungal infection in equine seems very challenging for owners and clinicians. The present study aimed to identify and characterize fungal species isolated from the eyes of clinically healthy and diseased equines (N = 100) from Dakahlia Governorate, Egypt. The work also involved morphological and molecular characterization of the major fungal species. In addition, correlations between the occurrence of isolated fungi and some of the potential risk factors were also investigated. Interestingly, the prevalence rate of ocular mycosis in all examined equines in the study was 28% and there were major clinical signs associated with ocular fungal infection. Moreover, the identified fungal species included *Aspergillus flavus*, *A. fumigatus*, *A. niger*, *Penicillium* spp., *Mucor* spp., and *Alternari* spp. with a corresponding prevalence rate of 63.9%, 27.8%, 15.3%, 18.1%, 13.9%, and 4.2%, respectively, in healthy equine eyes, while their prevalence in diseased equine eyes was 57.1%, 32.1%, 21.4%, 7.1%, 3.6%, and 0%. Furthermore, a statistical significant association (*p* < 0.05) was found between the frequency of isolation of *A. fumigatus* and *Penicillium* and several risk factors (breed, sex, and ground type), while the remaining risk factors and occurrence of fungi were not statistically correlated. A subset of the *Aspergillus* species samples positive by polymerase chain reaction (PCR) were sequenced and their phylogenetic analysis identified three species of *Aspergillus*. Taken together, our study provides novel data related to the occurrence of ocular mycosis in equine in Egypt. Given the zoonotic potential of some identified fungi, our data may be helpful for implementation of novel diagnostic and therapeutic strategies for combating this sight-threatening infection in equine.

## 1. Introduction

Ocular diseases and their related complications remain a serious health problem in equines worldwide [1]. These diseases have a great impact on the quality of life and value of affected horses, as well as their athletic and show potential or use for other purposes [1]. In addition, the pain associated with ocular diseases damages the animal welfare and may lead to partial or full blindness, which may result in exclusion of the horse from performance occupations and cause potential risk for the rider [2]. In fact, the ocular surface in horses, mules, and donkeys is naturally inhabited by microorganisms that constitute its normal microflora [3]. Scientists believe such flora play a defensive role against pathogenic microbes by depriving them from nutrients, producing antimicrobial substances, and occupying space on the corneal and conjunctival epithelium [4]. However, under particular disease conditions, the microbes composing the normal flora, including Gram-negative bacteria and fungi, may become opportunistic pathogens, [5,6]. Amongst others, 38% of infectious keratitis cases in equines caused by fungal organisms are believed to be seeded from the environment, thus seasonal variation could affect the equine conjunctival microflora [7,8]. Importantly, these opportunistic pathogens, which include a wide range of fungi and bacteria, can be transmitted to humans and cause potential health risks [9].

There is great variability in pathogenic fungal species and the visual outcome of ocular infection. Fungal infection of the eye is frequently encountered in horses but it is rare in ruminants, dogs, and cats [10]. The ubiquitous presence of fungi on the surface of the conjunctiva and cornea of equines, combined with suspected defects in the equine corneal immune system make corneal infection more likely to occur in equines than in other domestic animal species [10,11]. Furthermore, the progression of keratomycosis is favored by the use of topical corticosteroids and prolonged use of topical antibiotics [12,13]. Equine keratomycosis has been documented in various countries and regions worldwide including the USA [10,11,14,15,16], the United Kingdom [17], Germany [18], Australia [19], and Switzerland [20]. However, it should be stressed that fungal infection of the eye is more common in warm, humid environments such as the southern USA [12]. Fungal keratitis in horses is considered an infrequent problem in temperate regions of the world, where bacterial keratitis is the major problem [21,22]. In Brazil and the USA, fungi were detected most frequently in the eyes of animals that lived in stalls, where molds were more prevalent (91%) than yeasts [21]. This could mean that high humidity, dust and other hygienic problems in the stalls represent major risk factors for the development of keratomycosis in horses confined to stables [7]. Interestingly, the most common isolated fungal organisms from non-diseased eyes include *Cladosporium*, *Alternaria*, *Fusarium*, *Aspergillus* spp., and *Penicillium* spp. [7], while the most frequently reported fungal isolates from diseased equine eyes are *Aspergillus* spp., *Fusarium* spp., and *Candida* spp., which vary depending on the location [11,23]. Since there is a lack of information on the available epidemiological data related to the occurrence of keratomoycosis in equines in the Egyptian environment and the potential zoonotic risks of some of the frequently isolated fungal species, the present study was undertaken to investigate and characterize the occurrence of fungal pathogens isolated from equine eyes.

## 2. Material and Methods

### 2.1. Ethical Consideration

Ethical approval was obtained from a guidance of Research, Publication and Ethics of the Faculty of Veterinary Medicine, Mansoura University, Egypt, with code number R/31, which complies with all relevant Egyptian legislations in research and publications.

### 2.2. Study Area, Animals, and Medical Records

The study was carried out on a total number of 100 swabs from the eyes of clinically healthy and diseased equines (N = 100) from Dakahlia Governorate, Egypt from October 2018 to May 2019. This included 86 horses (61 clinically healthy and 25 with ophthalmic lesions) at a median age of 11.5 years old (range 0.5–22 years) and median weight of 330 kg (range 100–500 kg). In addition, 14 donkeys (*Equus asinus*) (11 clinically healthy and three with ophthalmic lesions) at a median age of 6.8 years (range 0.3–13 years) and at a median weight of 220 kg (range 80–350 kg) were also analyzed. The selected horses lived in semi-open stabling in an open yard from 08:00 till 14:00, then they were allowed to enter the closed pens. All horses were fed a concentrated diet and berseem in the winter and a concentrated diet and hay in the summer season. Importantly, horses and equine eyes were examined before application of any topical or systemic treatment. A questionnaire was given to managers at the time of visit, consisting of objective questions to obtain data regarding age, sex, breed, housing, season, hygiene, bedding, any stress factor related to the animal, and antimicrobial drugs used in the treatment of any previous ophthalmic infection. Importantly, the sampling process was carried out by a group of specialists and experts in the field of equine ophthalmology including clinicians, ophthalmologists, veterinary surgeons, and several experts from the Department of Infectious diseases and Internal Medicine and Clinical diagnosis, Faculty of Veterinary Medicine, Mansoura University, Egypt. The experts followed strict regulations during the sampling process to avoid violating any ethical boundaries. 

### 2.3. Clinical Examination and Sample Collection

Data concerned with the case history, clinical findings, and medical record for each horse and donkey were recorded. Ophthalmic history was obtained through a series of questions directed to the owners and farm personnel regarding the observed clinical signs. The clinical examination of the eye was performed without anesthetics for restraint purposes as it was a non-invasive study. The palpebral margins and the conjunctiva were examined for signs of inflammation, and the position of the third eyelid was evaluated. The opening of the palpebral fissure is readily achieved by using the thumb and forefinger from the same hand while approaching from the temporal aspect of the eye. Pupils were dilated with 1% atropine sulfate (atropine eye drops). The anterior chamber and posterior chamber of the eyes were examined with a direct ophthalmoscope. The clinical findings were recorded. Regarding sampling, a conjunctival swab from the inferior conjunctival fornix of both eyes was collected from each animal, without touching the eyelashes or eyelids, using a sterile swab under complete aseptic precautions. All samples were collected before the animals were given neither antibiotics (local or systemic) nor anesthetics as described elsewhere [24]. The collected swabs were directly placed in sterile test tubes containing tryptone soya broth (Oxoid, Oxoid Ltd., Cheshire, UK) as enrichment media and were sent directly to the laboratory of Internal Medicine, Faculty of Veterinary Medicine, Mansoura University, Egypt for further mycological examination.

### 2.4. Isolation and Identification of Fungal Species

All samples were incubated at 37 °C for 24 h, then a loopful was taken from each sample and streaked on Sabouraud dextrose agar (Oxoid) and incubated at 25 °C for isolation of fungi. Identification of fungal species was done by colony morphology and molecular characterization through sequencing of 18 S ribosomal RNA (rRNAs) gene.

### 2.5. Fungal DNA Extraction

The genomic DNA was extracted from 5–7 days old fungal cultures grown in malt extract agar media. The mycelial growth from the culture plate was scraped out using 2 mL of sterile distilled water. Two mL of spore suspension was then inoculated in a 100 mL yeast extract with supplements (YES) medium in a universal 250 mL flask and incubated with gentle shaking at 28 °C with 180 rpm for 48 h. Harvesting of the mycelia from the flasks was done by filtration under aseptic condition using a micro-cloth. The mycelia were then washed with sterile distilled water and placed in sterile Petri–dishes, stored at −20 °C overnight and freeze-dried. Later on, the freeze-dried mycelium was ground in a mortar using a sterile pestle and then placed in eppendorf tubes. DNA extraction was conducted using DNeasy kit (Qiagen, Hilden, Germany) according to the manufacturer’s instructions. To ensure the presence of fungal DNA, agarose gel electrophoresis was conducted and the DNA was visualized and imaged using the transilluminator of a gel documentation system (BIO-RAD, Gel Doc 2000, Budapest, Hungary).

### 2.6. Polymerase Chain Reaction (PCR)

Taq PCR Master mix (Qiagen) was used to amplify or synthesize DNA (genomic or plasmid) fragments that targets the 18 S ribosomal RNA (rRNAs) gene using a thermal cycler machine (Stratagene, San Diego, CA, USA) at the Regional Center for Mycology and Biotechnology Alazhar University, Cairo, Egypt. The primer set used was TCCGTAGGTGAACCTGCGG for the forward primer and TCCTCCGCT TAT TGA TAT GC for the reverse primer, to amplify a 595–600bp fragment [25]. The appropriate PCR conditions included a universal denaturation cycle (5 min at 94 °C), 30 cycles of annealing/extension reactions (20 s at 94 °C, 30 s at an optimum annealing temperature for each amplified fragment and 60 s at 72 °C and cycle of final extension step (5 min at 72 °C) followed by soaking at 4 °C.

### 2.7. Purification of DNA Fragments from the Gel

DNA fragments (PCR products and linear plasmid) were purified by agarose gel electrophoresis using the appropriate gel concentration stained with ethidium bromide according to the method described elsewhere [26].

### 2.8. DNA Sequencing, Assembling of Nucleotide Sequences, and Phylogenic Analysis

Some of positive PCR products belonged to *Aspergillus* fungi were directly purified then sequenced using the Cy5/Cy5.5 Dye Primer Sequencing kit (Visible Genetics Inc., Toronto, ON, Canada) with the Open Gene automated DNA sequencing system [27,28] at the Regional Center For Mycology and Biotechnology, Alazhar University, Cairo, Egypt. The resulting nucleotide sequences were subjected to BLAST algorithms and databases from the National Center for Biotechnology NCBI (http://www.ncbi.nlm.nih.gov/html) to compare the resulting sequence to those available in GenBank to confirm PCR specificity. Molecular evolutionary genetics analysis (MEGA) MEGA X (http://www.megasoftware.net/) software was also used for sequence analysis and alignments [29]. The phylogram is drawn to scale, with branch lengths (next to the branches) in the same units as those of the evolutionary genetic distances (0.05%) [30].

### 2.9. Statistical Analysis

The statistical analysis was performed on SPSS 21 (IBM, Armonk, New York, NY, USA). Numerical data were expressed as median (range), while categorical data were expressed as number (%). Chi-square tests were conducted to assess the correlation between various risk factors, including age, sex, breed, housing, season, hygiene, ground type, stress and frequency of isolation (percentage) of different fungal species from ocular swabs. The *p*-value, odds ratio (OR), and 95% confidence interval (CI 95%) were recorded as a multivariable logistic regression model to detect the associated risk factors of infection. For all statistical analysis, variables at *p*-value < 0.05 were considered to be significant.

## 3. Results

### 3.1. Clinical Signs

**Figure 1:**
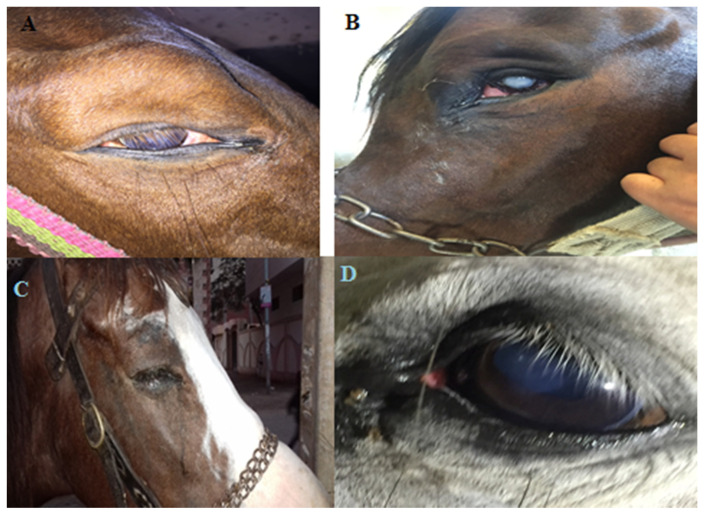
Clinical signs suggest ophthalmic infection. (**A**) A horse with ocular infection showing corneal opacity and mucopurulent discharge. (**B**) A horse’s eye, following exposure to ocular trauma showing corneal opacity. (**C**) A horse suffering from conjunctivitis showing blepharospasm, eyelid edema and continuous lacrimation. (**D**) A donkey showing mucopurulent discharge with eyelid edema.

Out of the 100 animals investigated (86 horses and 14 donkeys), 25 horses and three donkeys (28 in total) developed one or more of the following clinical signs that suggest ophthalmic infection: conjunctival hyperemia, continuous lacrimation, blepharospasm, eye lid edema, mucopurulent ocular discharge, corneal edema, and corneal opacity, examples of which are shown in Figure 1. There were no behavioral or systemic signs of disease in all the studied group. Furthermore, all the parameters of heart rate, respiratory rate, and rectal temperature were within the normal range. 

### 3.2. Morphology of the Isolated Fungal Species

The colonies of the different species of fungi started to be visible after 4–5 days of cultivation on Sabouraud dextrose agar media. The morphology and characters of the different species of fungi are summarized in Table 1.

### 3.3. Molecular Identification, Sequence Analysis, and Phylogeny of Isolated Fungi

Regarding the molecular identification, the amplified DNA fragment size for the targeted genes (18S rRNA) extracted from the colonies revealed an amplicon of approximately 595–600 base pair (bp). The gene sequences and phylogenetic analysis showed that the pair wise comparison illustrates the low distance between the sequences from selected three fungal species of *Aspergillus* compared with reference sequences from GenBank which is shown in Supplementary Figures S1–S3. In this concern, as shown in Supplementary Figure S1, the percentage identity between the *Aspergillus flavus* sequence of our study and previous sequences in GeneBank was 100% with *A. flavus* isolate RF-02 S rRNA gene (accession no. KY933394.1) and *A. flavus* strain RF-03 S rRNA gene (accession no. MF120213.1), while the identity was 99.83% with *A. flavus* isolate SF53 S rRNA gene (accession no. MG976497.1) and *A. flavus* strain CBS 126855 S rRNA gene (accession no. MH864264.1).

Regarding *Aspergillus fumigatus,* as predicted in Supplementary Figure S2, the percentage of identity between the *A. fumigatus* sequence obtained in our present work was 99.8% with *A. fumigatus* strain FJAT-31052 S rRNA (accession no. KU687812.1), while it showed 99.59% identity with the following strains; *A. fumigatus isolate* EGDA31 S rRNA (accession no. MH591451.1), *A. fumigatus* isolate 1052 18S S rRNA (accession no. KT826617.1), *A. fumigatus* strain IHEM 18963 isolate ISHAMITS_ID MITS169 18S rRNA (accession no. KP131566.1), *A. fumigatus* strain HQ 18S rRNA (Accession no. EU139476.1), and *A. fumigatus* isolate X 7 18S rRNA (accession no. KJ958365.1). On the other hand, there was 100 percentage identity between the obtained sequences of *Aspergillus niger* and previous sequences in GeneBank, *A. niger* strain RAF106 S rRNA (accession no. MN195121.1), *A. niger* isolate KUASR15 S rRNA (accession no. MN187307.1), *A. niger* isolate T8 internal transcribed spacer 1 (ITS) (accession no. MN180811.1), *A. niger* strain 22 ITS1 (accession no. MN133869.1), and *A. niger* isolate SVUA ITS1 (accession no. MK258199.1).

### 3.4. Frequency of Isolated Fungi between Healthy and Diseased Equines’ Eyes and Associated Risk Factors

As depicted in Table 2, *Aspergillus flavus* was isolated from 63.9% of healthy eyes and 57.1% of diseased eyes while *Aspergillus fumigatus* was isolated from 27.8% of healthy eyes and from 32.1% of diseased eyes. In addition, *Aspergillus niger* was isolated from 11 (15.3%) healthy animals and six (21.4%) animals with diseased eyes, while *Mucor* species was isolated from 13 (18.1%) healthy eyes and two (7.1%) diseased eyes. Meanwhile, *Penicillium* species were isolated from 10 (13.9%) healthy eyes and one (3.6%) diseased eye while *Alternaria* species was isolated from three (4.2%) healthy eyes but was not isolated from any diseased eyes. Collectively, *Aspergillus flavus*, *Aspergillus fumigatus*, *Aspergillus niger*, *Mucor* species, *Penicillium* species and *Alternaria* species were the most commonly isolated fungal species from healthy and diseased equine eyes; however, there was no statistical significant difference in the frequency of isolated fungi between healthy and diseased equine eyes.

Table 3, Table 4, Table 5, Table 6, Table 7 and Table 8 show the correlation between the variable studied risk factors and the frequency of isolated species of fungi in healthy and diseases eyes of examined animals. As mentioned above, the studied risk factors included age, sex, breed, housing, season, hygiene, ground type, and other stress factors. Among others, there was a significant association noted between the frequency of the isolation of *Aspergillus fumigatus* from eyes and both the breed and ground type (both *p*-value = 0.025; OR = 0.337; 99% CI: 0.260–0.437). In this concern, the frequency of isolation of *Aspergillus fumigatus* was considerably higher in Arabian horses than other breeds and donkeys, where this fungus was isolated from 16 (55.2%) Arabian horses versus 13 (44.8%) horses from the other breeds and donkeys (Table 4). In addition, *Aspergillus fumigatus* was isolated at significantly higher levels from animals housed on concrete flooring (55.2%) compared with mud flooring (44.8%) (Table 4). Also, there was a significant association between *Penicillium* spp. isolation and the following risk factors, sex (*p* = 0.021) and housing (*p* = 0.024) (Table 7). With respective to the sex of the animal, *Penicillium* was only isolated in females (100%) (Table 7). Meanwhile for housing, the percentage of isolated *Penicillium* was higher in mixed housing (90.9%) in comparison with indoor systems (9.1%) (Table 7). On the other hand, there were no statistical significance association noted between *Aspergillus flavus*, *Aspergillus niger*, *Mucor* spp., *Alternaria* spp. and all the mentioned risk factors (Table 3, Table 4, Table 5, Table 6, Table 7 and Table 8).

## 4. Discussion

The corneal epithelium and tear film of the eyes represent a major barrier for protection of equine eyes from many contaminates and from invasion by pathogens that include bacteria and fungi [31]. The physiology of the equines eye may contribute in potentiating their susceptibility to infection [31,32]. For example, the large size of the eye, the prominence and unprotected position of the eye, and the tear film instability of equine eyes expose them to environmental factors such as trauma of the cornea with vegetative materials, organic matter, head collar, and contaminated soils [15,31,32]. These factors, which either alone or in association with other factors, represent leading causes of keratomycosis as they destruct the main barriers, favoring pathogen entry [33]. Among other pathogens, fungal infection represents one of sight-threatening disease in different animals species, but equines seem most frequently affected due to the lateral location of their eyes [34,35]. Previous studies have shown that fungi were isolated from normal, healthy eyes of cows (100%), horses (12%), dogs (22%), cats (40%), and sheep (86%) [8,36]. The conjunctiva of theses animal species were colonized by a subset of fungal species including *Aspergillus* spp. in 12% of cows, 56% of horses, and 8% of the cats, but not detected in dogs. *Penicillium* spp. and *Cladosporium* spp. were isolated ubiquitously from these species [8]. Moreover, five species of fungi were isolated from normal sheep conjunctiva, including *Mucor* spp., *Aspergillus* spp., *Penicillium* spp., *Alternaria* spp., and *Cladosporium* spp. [36].The present study provides novel data related to the occurrence of different species of fungi isolated from healthy and diseased eyes of equines in Dakahlia governorate, Egypt. To achieve this objective, the work involved morphological identification and molecular characterization of the major isolated fungi combined with an assessment of some possible risk factors that might influence the epidemiological pattern of the disease. This seems to be the first study involving isolation and identification of different mycosis from the eyes of equines in Egypt.

In accordance with its pathogenesis, keratomycosis is considered a subset of eye infection which is associated with primary disruption of the corneal epithelium and responsible for binding of environmental organisms to the corneal stroma [32,37]. Upon their binding, the fungal hyphae release proteases that inhibit the angiogenesis in susceptible cornea, corneal melting and anterior uveitis [37]. The hallmark of mycotic keratitis in horses is the induction of rapidly and progressive ulceration and perforation on the cornea [38]. More importantly, some of these major leading ubiquitous organisms causing keratomycosis may be of potential zoonotic concern, e.g., *Aspergillus* spp., and therefore may be occupation risks for agricultural workers and farmers [39]. Clearly, early identification of the causative fungi is considered a cornerstone for designing control strategies. At the same time, the identification of the causative agent represent a major challenge for proper therapeutic intervention and consequently for salvage of the vision [32]. The prevalence of keratomycosis or ocular mycosis in equines has been documented in some previous studies worldwide where the diagnosis in these studies was carried out using either one or often a combination of the following methods: cytology, culture, histopathology, and molecular identification [11,14,23,40,41,42,43,44]. In accordance with the reported clinical signs, the majority of infected horses and donkeys experienced one or more of the various clinical signs that include conjunctival hyperemia, continuous lacrimation, blepharospasm, eye lid edema, and mucopurulent ocular discharge. Taken into account, these clinical signs are not specific for certain species of fungi but similar clinical signs were recorded in previous reports with equine ulcerative fungal keratitis [45,46].

In our present results, the prevalence of kertaomycosis was 28% in relation to the total number of the examined ophthalmic cases. Interestingly, this prevalence is higher than that reported in several previous studies [17,40]. In a previous study carried out by McLaughlin et al. (1992) [47], the reported prevalence of keratomycosis was 10%. Furthermore, other studies have reported prevalence rates of 8.62% and 7%, respectively, which are lower than our current findings [40,48]. This may relate to the fact that a higher number of equines were examined in our study versus the majority of the previous reports worldwide [17,40]. Alternatively, the difference in the prevalence of fungal infection compared to our study might be attributed to the hypothesis that the seasonal variation and temperature conditions, housing and management conditions might influence the epidemiological pattern of keratomycosis [35,49]. Along the same line, equine fungal keratitis is usually recorded in warm and humid climate regions, which suggest that seasonal variation is an important factor influencing the occurrence of the disease [31,50,51]. However, there is raised awareness of its prevalence in subtropical areas and during summer and autumn months with sufficient temperature and precipitation [12,43,50,52]. Clearly, the difference in geographic location might favor the existence of certain species of fungi in specific regions than others [17,21,44]. Our present results showed isolation of six major species of fungi from healthy and diseased equine eyes. These fungal species are *Aspergillus flavus*, *Aspergillus fumigatus*, *Aspergillus niger*, *Penicillium* spp., and *Mucor* spp. Furthermore, a sixth species, *Alternari* spp., was only isolated from the healthy eyes of examined animals. Given the little difference on the frequency and the prevalence of the isolated fungi, *Aspergillus flavus*, *Aspergillus fumigatus*, and *Aspergillus niger* were the most common etiological agents have been isolated from eyes of healthy and diseased equine, which is consistent with several previous studies [35,40,41,43]. Similar studies revealed that *Aspergillus* and *Fusarium* counts for one-third of all major causes of traumatic keratitis [35,40,49], while some other fungal genera could be isolated such as *Candida*, *Penicillium*, and *Alternaria* [11,14,23,42]. In the same line, several previous works concluded that mixed infections of *Aspergillus* and *Pseudomonas* spp. in horses represent the major isolated pathogens in severe corneal diseases [3,16].

Analysis of the data presented here revealed that there are several predisposing factors such as; breed, age, sex, species, season, and stabling that might influence the epidemiological pattern of keratomycosis [14]. However, it should be borne in mind that the small number of clinical cases in our present study makes it difficult to study the correlation of predisposing (risk) factors to the occurrence of the disease, and therefore, it might influence the significance of the results of the statistical analysis difference or not as documented elsewhere. As shown in Table 2, the percentage of isolation from fungi was higher in healthy equines than diseased equines, which is consistent with several previous studies [35,53]. The occurrence of *Aspergillus fumigatus* was higher in Arabian horses (55.2%) in comparison with other horse breeds and donkeys (44.8%) that implicate the breed as a possible predisposing factor for fungal infection of equine eyes (Table 2). Similar results were reported in previous studies which found that the occurrence of keratomycosis affected some horse breeds more than others [12]. Furthermore, the highest percentage of isolation of *Aspergillus fumigatus* was recorded in animals stabled with concrete flooring (55.2%) compared with those stabled with mud flooring (44.8%) (Table 4). On the other hand, there was a significant association between the frequency of the isolation of *Penicillium* and both the sex of the animal and housing factors. In this concern, there was correlation between the positivity to *Penicillium* from eyes of infected equids and the sex; where more female equids were infected than male equids. As depicted in Table 7, for housing, the isolation percentage of *Penicillium* from animals kept indoors or outdoors was 9.1% versus 90.9%, respectively. This finding is in line with previous studies from USA and Brazil that reported a higher occurrence of fungi isolated from the eyes of stabled equids compared to those horses living outside [7,21,54]. These data reflects that the high humidity, dust, and other hygienic problems in the stables represent an important risk factor for keratomycosis in equids confined to stables [7]. However, the majority of outdoors living donkeys in rural areas were found to harbor fungi in their eyes [55]. It should be stressed that it is very hard to compare the prevalence of ocular fungal infection of horses and donkeys living outside versus stabled ones because of their peculiar kind of husbandry, especially those from rural areas and kept outdoors all day. In Tuscany, stabled horses with increased levels of humidity and dust were more frequently positive to kertaomycosis than those living outside [56], leading to a higher risk of ocular contamination by fungi [21,56].

Meanwhile, for the other risk factors and the remaining fungal species, despite the existence of differences between the frequency of isolation of fungi and risk factors, no significant correlation was reported, which is consistent with several previous studies [14,57,58]. Furthermore, there was no correlation and no statistical significant difference between the other studied possible risk factors and the frequency of the isolation of the other isolated fungal species, which is consistent with the hypothesis that keratomycosis can affect any horse and has no breed, age, or sex predisposition, but geographic location and housing and management practices might influence the epidemiology of the disease [14,57,58]. The role of molecular methods in detection of keratomycososis in equine has been documented in several previous studies [59]. In our present results, the amplified DNA fragment size target 18S rRNA gene from the colonies revealed an amplicon of 594–600 bp in size, which is similar to that was reported elsewhere [60]. It is worth mentioning that our present work investigated the molecular identity of the major isolated species of fungi that were *Aspergillus flavus, Aspergillus fumigatus,* and *Aspergillus niger*. In addition, the data were then confirmed illustrated through a phylogenetic analysis for three isolated of three *Aspergillus* species and this might reveal the wide spread of these genotypes to Egypt.

## 5. Conclusions

Given the findings, our study reveals high prevalence of fungal infection in the investigated equine eyes from Dakahlia governorate, Egypt and provides novel information that should turn our attention toward the importance of early recognition of the disease agent for the prompt intervention and successful management of the case. Our study also proposes further future research to explore more about fungal mycotic infection in different animal species together with investigation of the occurrence of ocular mycosis in people who are in contact with these infected animals. Other alternative prophylactic strategies, good management practices, and periodical surveillance regimes may also be helpful to reduce the risk of infection.

## Figures and Tables

**Table 1:** The morphology and characters of colonies on agar, the colonies of the different species of fungi, and their appearance on Sabouraud dextrose agar media (SDA).

Fungal Species	Appearance on SDA
*Asp. flavus*	Colonies were greenish-yellow to olive and some had a white border. Texture is often floccose, especially near the center and overall can be velvety to woolly.
*Asp. fumigatus*	Colonies appeared blue-green, powdery.
*Asp. niger*	The initial growth of *Aspergillus niger* is white, becoming black later, giving “salt and pepper appearance,” which results from darkly pigmented conidia borne in large numbers on conidiophores.
*Mucor species*	Colonies appeared cottony to fluffy, white to yellow, becoming dark grey, with the development of sporangia.
*Penicillium species*	Colonies were fast growing, in shades of green, sometimes white, mostly consisting of a dense felt of conidiophores.
*Alternaria species*	Colonies appeared black to olivaceous-black or greyish and are suede-like to floccose.

**Table 2:** The frequency of isolated fungal species between healthy and diseased equine eyes.

	Healthy (n = 72)	Diseased (n = 28)	Odds Ratio	*p*-Value	CI 95%
Aspergillus flavus			1.327	0.344	0.545–3.230
Positive	46(63.9%)	16(57.1%)			
Negative	26(36.1%)	12(42.9%)			
Aspergillus fumigatus			0.812	0.420	0.315–2.091
Positive	20(27.8%)	9(32.1%)			
Negative	52(72.2%)	19(67.9%)			
Aspergillus niger			0.661	0.322	0.218–2.002
Positive	11(15.3%)	6(21.4%)			
Negative	61(84.7%)	22(78.6%)			
Mucor species			2.864	0.143	0.603–13.611
Positive	13(18.1%)	2(7.1%)			
Negative	59(81.9%)	26(92.9%)			
Penicillium species			4.355	0.127	0.531–35.731
Positive	10(13.9%)	1(3.6%)			
Negative	62(86.1%)	27(96.4%)			
Alternaria species			-	0.369	0.627–0.807
Positive	3(4.2%)	0(0%)			
Negative	69(95.8%)	28(100%)			

**Table 3:** Risk factors of *Aspergillus flavus*.

	*Aspergillus Flavus*		Odds Ratio	*p*-Value	CI 95%
	Negative	Positive			
Age			1.415	0.357	0.488–4.105
<5	32(84.2%)	49(79%)			
5–10	6(15.8%)	13(21%)			
Breed			0.521	0.126	0.203–1.334
Arabian	30(78.9%)	41(66.1%)			
Others	8(21.1%)	21(33.9%)			
Sex			0.560	0.163	0.218–1.440
Male	8(21.1%)	20(71.4%)			
Female	30(78.9%)	42(58.3%)			
Housing			0.560	0.128	0.240–1.308
Indoor	12(31.6%)	28(45.2%)			
Mixed	26(68.4%)	34(54.8%)			
Season			2.000	0.095	0.823–4.861
Winter	14(36.8%)	14(22.6%)			
Spring	24(63.2%)	48(77.4%)			
Hygiene			0.924	0.591	0.252–3.394
Good	4(10.5%)	7(11.3%)			
Bad	34(89.5%)	55(88.7%)			
Ground type			0.521	0.126	0.203–1.334
Mud	8(21.1%)	21(33.9%)			
Concrete	30(78.9%)	41(66.1%)			
Stress			0.645	0.219	0.271–1.536
Individual	11(28.9%)	24(38.7%)			
Over-crowding	27(71.1%)	38(61.3%)			

**Table 4:** Risk factors of *Aspergillus fumigatus.*

	*Aspergillus Fumigatus*		Odds Ratio	*p*-Value	CI 95%
	Negative	Positive			
Age			1.564	0.284	0.546–4.484
<5	59(83.1%)	22(75.9%)			
5–10	12(16.9%)	7(24.1%)			
Breed			0.358	0.025	0.143–0.898
Arabian	55(77.5%)	16(55.2%)			
Others	16(22.5%)	13(44.8%)			
Sex			1.721	0.215	0.615–4.819
Male	22(31%)	6(20.7%)			
Female	49(69%)	23(79.3%)			
Housing			0.507	0.097	0.211–1.218
Indoor	25(35.2%)	15(51.7%)			
Mixed	46(64.8%)	14(48.3%)			
Season			1.320	0.386	0.490–3.558
Winter	21(29.6%)	7(24.1%)			
Spring	50(70.4%)	22(75.9%)			
Hygiene			1.960	0.326	0.397–9.681
Good	9(12.7%)	2(6.9%)			
Bad	62(87.3%)	27(93.1%)			
Ground type			0.358	0.025	0.143–0.898
Mud	16(22.5%)	13(44.8%)			
Concrete	55(77.5%)	16(55.2%)			
Stress			0.450	0.062	0.185–1.095
Individual	21(29.6%)	14(48.3%)			
Over-crowding	50(70.4%)	15(51.7%)			

**Table 5:** Risk factors of *Aspergillus niger.*

	*Aspergillus niger*		Odd Ratio	*p*-Value	CI 95%
	Negative	Positive			
Age			1.395	0.410	0.399–4.880
<5	68(81.9%)	13(76.5%)			
5–10	15(18.1%)	4(23.5%)			
Breed			1.401	0.411	0.416– 4.720
Arabian	58(69.9%)	13(76.5%)			
Others	25(30.1%)	4(23.5%)			
Sex			0.661	0.322	0.218–2.002
Male	22(26.5%)	6(35.3%)			
Female	61(73.5%)	11(64.7%)			
Housing			1.750	0.242	0.565–5.421
Indoor	35(42.2%)	5(29.4%)			
Mixed	48(57.8%)	12(70.6%)			
Season			0.920	0.549	0.292–2.902
Winter	23(27.7%)	5(29.4%)			
Spring	60(72.3%)	12(70.6%)			
Hygiene			2.192	0.406	0.262–18.363
Good	10(12%)	1(5.9%)			
Bad	73(88%)	16(94.1%)			
Ground type			1.401	0.411	0.416–4.720
Mud	25(30.1%)	4(23.5%)			
Concrete	58(69.9%)	13(76.5%)			
Stress			1.358	0.408	0.437–4.228
Individual	30(36.1%)	5(29.4%)			
Over-crowding	53(63.9%)	12(70.6%)			

**Table 6:** Risk factors of *Mucor*.

	*Mucor* Species		Odd Ratio	*p*-Value	CI 95%
	Negative	Positive			
Age			1.078	0.578	0.272–4.273
<5	69(81.2%)	12(80%)			
5–10	16(18.8%)	3(20%)			
Breed			0.556	0.235	0.178–1.737
Arabian	62(72.9%)	9(60%)			
Others	23(27.1%)	6(40%)			
Sex			0.524	0.206	0.167–1.640
Male	22(25.9%)	6(40%)			
Female	63(74.1%)	9(60%)			
Housing			0.383	0.078	0.124–1.177
Indoor	31(36.5%)	9(60%)			
Mixed	54(63.5%)	6(40%)			
Season			1.667	0.342	0.433–6.419
Winter	25(29.4%)	3(20%)			
Spring	60(70.6%)	12(80%)			
Hygiene			0.770	0.518	0.149–3.973
Good	9(10.6%)	2(13.3%)			
Bad	76(89.4%)	13(86.7%)			
Ground type			0.556	0.235	0.178–1.737
Mud	23(27.1%)	6(40%)			
Concrete	62(72.9%)	9(60%)			
Stress			0.777	0.434	0.252–2.395
Individual	29(34.1%)	6(40%)			
Over-crowding	56(65.9%)	9(60%)			

**Table 7:** Risk factors of *Penicillium*.

	*Penicillium* Species		Odd Ratio	*p*-Value	CI 95%
	Negative	Positive			
Age			0.394	0.338	0.047–3.285
<5	71(79.8%)	10(90.9%)			
5–10	18(20.2%)	1(9.1%)			
Breed			4.590	0.113	0.560–37.625
Arabian	61(68.5%)	10(90.9%)			
Others	28(31.5%)	1(9.1%)			
Sex			-	0.021	1.070–1.302
Male	28(31.5%)	0(0%)			
Female	61(68.5%)	11(100%)			
Housing			7.800	0.024	0.957–63.559
Indoor	39(43.8%)	1(9.1%)			
Mixed	50(56.2%)	10(90.9%)			
Season			4.355	0.127	0.531–35.731
Winter	27(30.3%)	1(9.1%)			
Spring	62(69.7%)	10(90.9%)			
Hygiene			-	0.258	1.055–1.234
Good	11(12.4%)	0(0%)			
Bad	78(87.6%)	11(100%)			
Ground type			4.590	0.113	0.560–37.625
Mud	28(31.5%)	1(9.1%)			
Concrete	61(68.5%)	10(90.9%)			
Stress			6.182	0.051	0.757–50.463
Individual	34(38.2%)	1(9.1%)			
Over-crowding	55(61.8%)	10(90.9%)			

**Table 8:** Risk factors of *Alternaria.*

	*Alternaria* Species		Odd Ratio	*p*-Value	CI
	Negative	Positive			
Age			-	0.528	0.923–1.005
<5	78(80.4%)	3(100%)			
5–10	19(19.6%)	0(0%)			
Breed			0.193	0.201	0.017–2.215
Arabian	70(72.2%)	1(33.3%)			
Others	27(27.8%)	2(66.7%)			
Sex			0.771	0.631	0.067–8.861
Male	27(27.8%)	1(33.3%)			
Female	70(72.2%)	2(66.7%)			
Housing			0.322	0.351	0.028–3.676
Indoor	38(39.2%)	2(66.7%)			
Mixed	59(60.8%)	1(33.3%)			
Season			-	0.369	0.994–1.095
Winter	28(28.9%)	0(0%)			
Spring	69(71.1%)	3(100%)			
Hygiene			1.035	0.702	0.995–1.076
Good	11(11.3%)	0(0%)			
Bad	86(88.7%)	3(100%)			
Ground type			0.193	0.201	0.017–2.215
Mud	27(27.8%)	2(66.7%)			
Concrete	70(72.2%)	1(33.3%)			
Stress			0.258	0.280	0.023–2.949
Individual	33(34%)	2(66.7%)			
Over-crowding	64(66%)	1(33.3%)			

## Supplementary Materials

The following are available online at https://www.mdpi.com/2306-7381/7/3/130/s1.
