# Supplementary material for: Epidemiological and Molecular Investigation of Ocular Fungal Infection in Equine from Egypt
Source: Vet Sci. 2020 Sep 8;7(3):130. doi: 10.3390/vetsci7030130 (PMC7558555; doi:10.3390/vetsci7030130)
Supplement: Supplementary file 1 [file vetsci-07-00130-s001.zip › vetsci-905302-supplementary/Supplementary Figures/Supplementary Figures.docx]

Article

Epidemiological and Molecular Investigation of Ocular Fungal Infection in Equine from Egypt

Amin Tahoun ^1,†^, Helmy K. Elnafarawy ^2,†^, Ehab Kotb Elmahallawy ^3,4,^*, Abdelhamed Abdelhady ^5^, Amira M. Rizk ^6^, Hanem El-Sharkawy ^7^, Mohamed A. Youssef ^2^, Sabry El-Khodery ^2^, and Hussam M. M. Ibrahim ^2,^*

^1^ Department of Animal Medicine, Faculty of Veterinary Medicine, Kafrelshkh University, Kafrelsheikh 33511, Egypt; amin12_veta@yahoo.com

^2^ Department of Internal Medicine and Infectious Diseases, Faculty of Veterinary Medicine, Mansoura University, Mansoura 35516, Egypt; [helmykamal@mans.edu.eg](mailto:helmykamal@mans.edu.eg) (H.K.E.); mohamed.youssef @mans.edu.eg (M.A.Y.) ; khodery@mans.edu.eg (S.E.-K.)

^3^ Department of Zoonoses, Faculty of Veterinary Medicine, Sohag University, Sohag 82524, Egypt

^4^ Department of Biomedical Sciences, University of Leon, s/n, 24071 León, Spain

^5^ Parasitology and Animal Diseases, National Research center, Dokki, Giza, 12622, Egypt; [afanrc@yahoo.com](mailto:afanrc@yahoo.com)

^6^ Department of Bacteriology, Mycology and Immunology, Faculty of Veterinary Medicine, Benha University, Benha 13518, Egypt; dr_az80@yahoo.com

^7^ Department of Poultry and Rabbit Diseases, Faculty of Veterinary Medicine, Kafrelsheikh University, Kafrelsheikh 33511, Egypt; hanem_amin@yahoo.com

***** Correspondence: eehaa@unileon.es (E.K.E.); dr_hussamhabosha@yahoo.com (H.M.M.I.)

^†^ These authors are contributed equally in the manuscript.


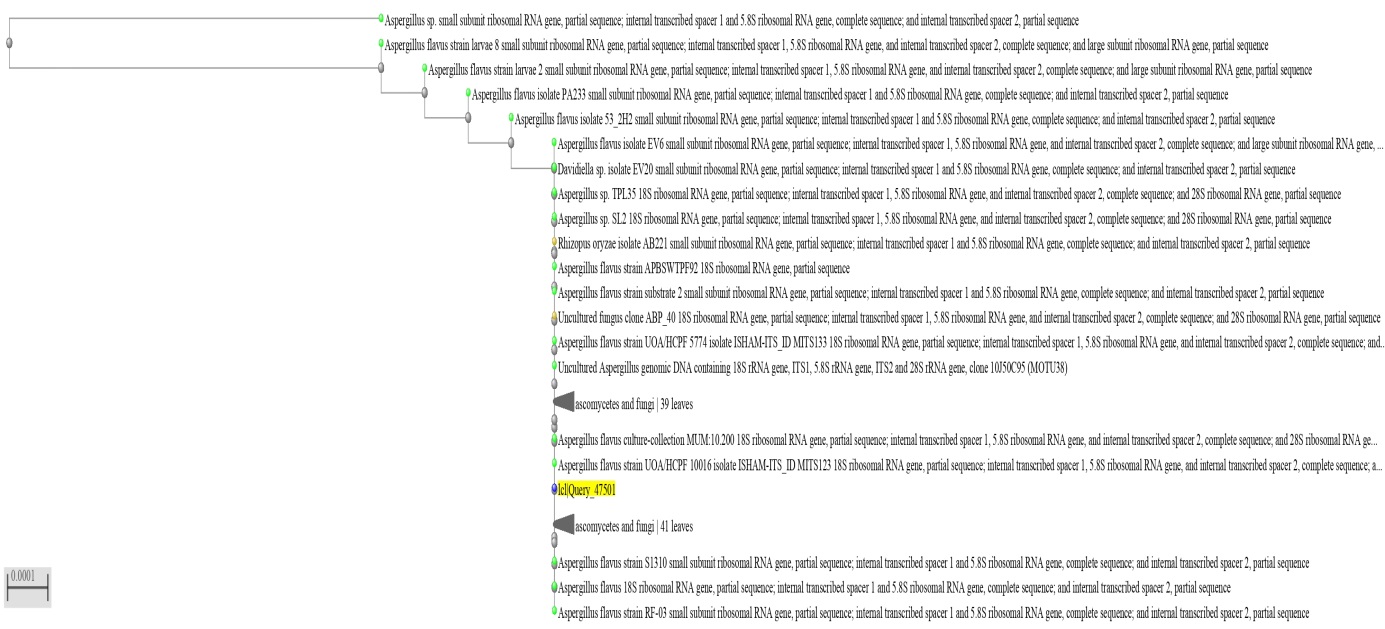
**Supplementary Figure 1**. Phylogenetic analysis and evolutionary relationship of the *Aspergillus flavus* nucleotide sequences isolated from ocular swabs of equine (ICI Query_47501) compared with reference sequences from GenBank based on the *18S rRNA* gene. The tree and the neighbor-joining analysis (NJ) were constructed in MEGA X software using *Aspergillus flavus* as out group, with genetic distance of 0.001. The resulting sequence of *Aspergillus flavus* was identical to previous sequences in Gene bank such as *Aspergillus flavus* isolate RF-02 S rRNA gene (Accession no. KY933394.1) and *Aspergillus flavus* strain RF-03 S rRNA gene (Accession no. MF120213.1).


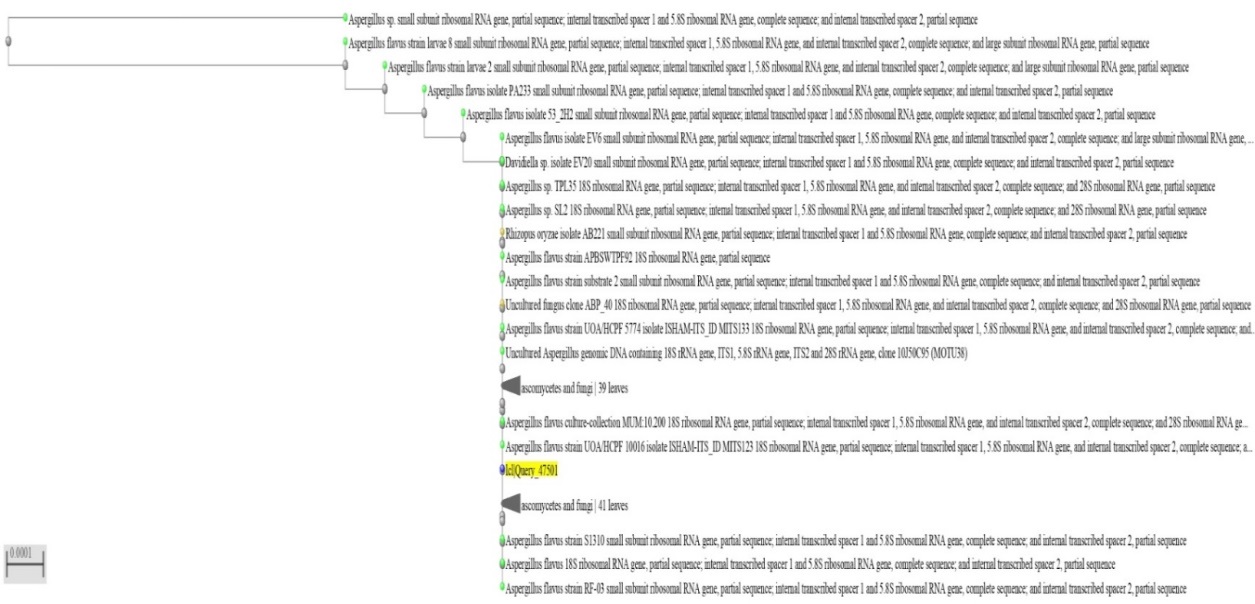


**Supplementary Figure 2.** Phylogenetic analysis and evolutionary relationship of the *Aspergillus fumigatus* nucleotide sequences isolated from ocular swabs of equine (ICI Query_47501) compared with reference sequences from GenBank based on the *18S rRNA* gene. The tree and the neighbor-joining analysis (NJ) were constructed in MEGA X software using *Aspergillus fumigatus* as out group, with genetic distance of 0.001. The resulting sequence of *Aspergillus fumigatus* was closely similar to previous sequences in Gene bank such as *Aspergillus fumigatus* strain FJAT-31052 S rRNA (Accession no. KU687812.1), *Aspergillus fumigatus isolate* EGDA31 S rRNA (Accession no. MH591451.1) and *Aspergillus fumigatus* strain HQ 18S rRNA (Accession no. EU139476.1).


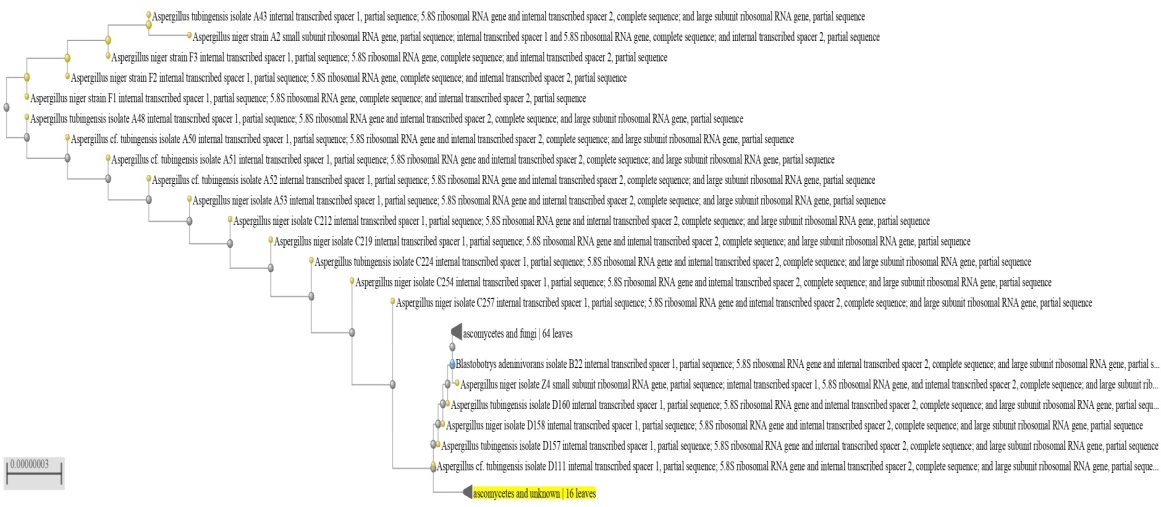


**Supplementary Figure 3.** Phylogenetic analysis and evolutionary relationship of the *Aspergillus niger* nucleotide sequences isolated from ocular swabs of equine (ICI Query_47501) compared with reference sequences from GenBank based on the *18S rRNA* gene. The tree and the neighbor-joining analysis (NJ) were constructed in MEGA X software using *Aspergillus niger* as out group, with genetic distance of 0.00000003. The resulting sequence of *Aspergillus niger* was closely similar to previous sequences in Gene bank such as *Aspergillus niger* strain RAF106 S rRNA (Accession no. MN195121.1) and isolate KUASR15 S rRNA (Accession no. MN187307.1).
