# Supplementary figures and images for: Epidemiological and Molecular Investigation of Ocular Fungal Infection in Equine from Egypt
Source: Vet Sci. 2020 Sep 8;7(3):130. doi: 10.3390/vetsci7030130 (PMC7558555; doi:10.3390/vetsci7030130)

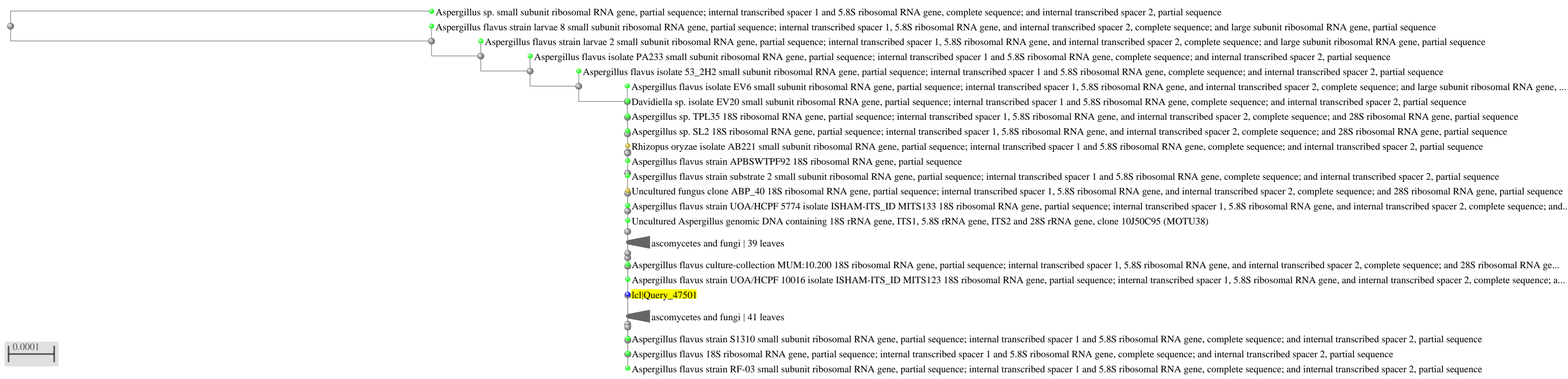

Supplement: Supplementary file 1 [file vetsci-07-00130-s001.zip › vetsci-905302-supplementary/Supplementary Figures/Figure 1 A.flavus Tree.pdf]

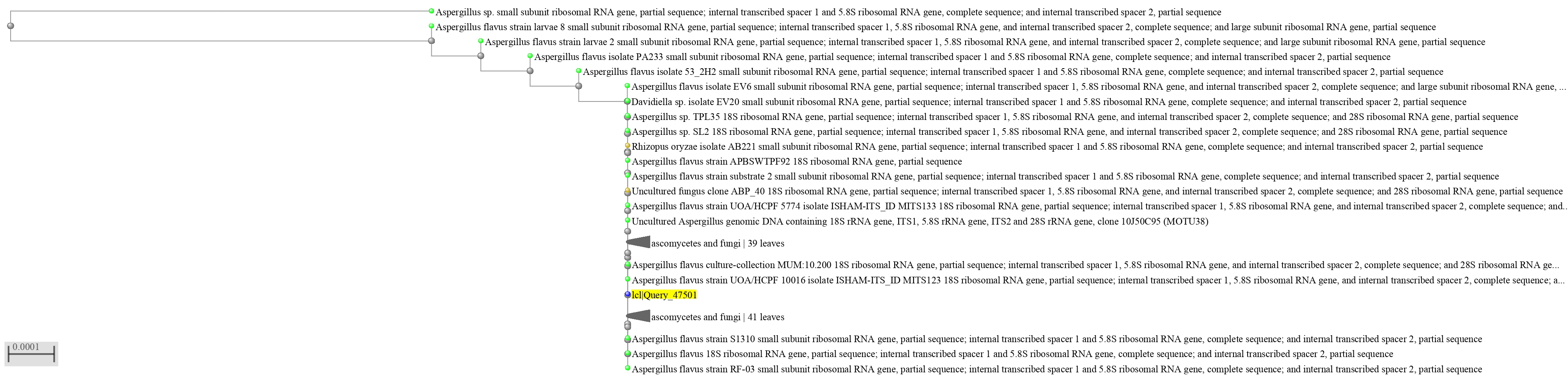

Supplement: Supplementary file 1 [file vetsci-07-00130-s001.zip › vetsci-905302-supplementary/Supplementary Figures/Figure 1.jpg]

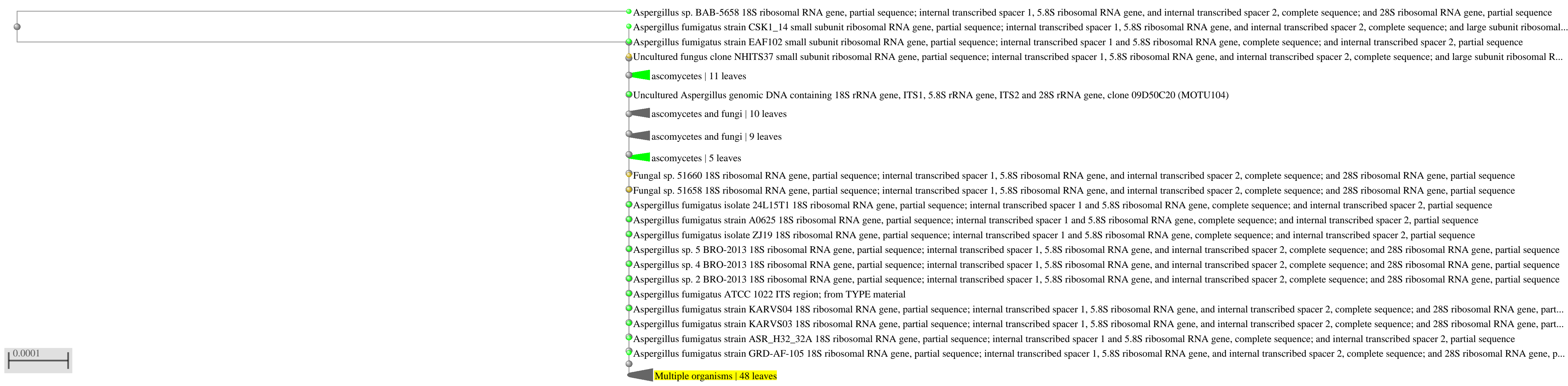

Supplement: Supplementary file 1 [file vetsci-07-00130-s001.zip › vetsci-905302-supplementary/Supplementary Figures/Figure 2 A.fumigatus Tree.pdf]

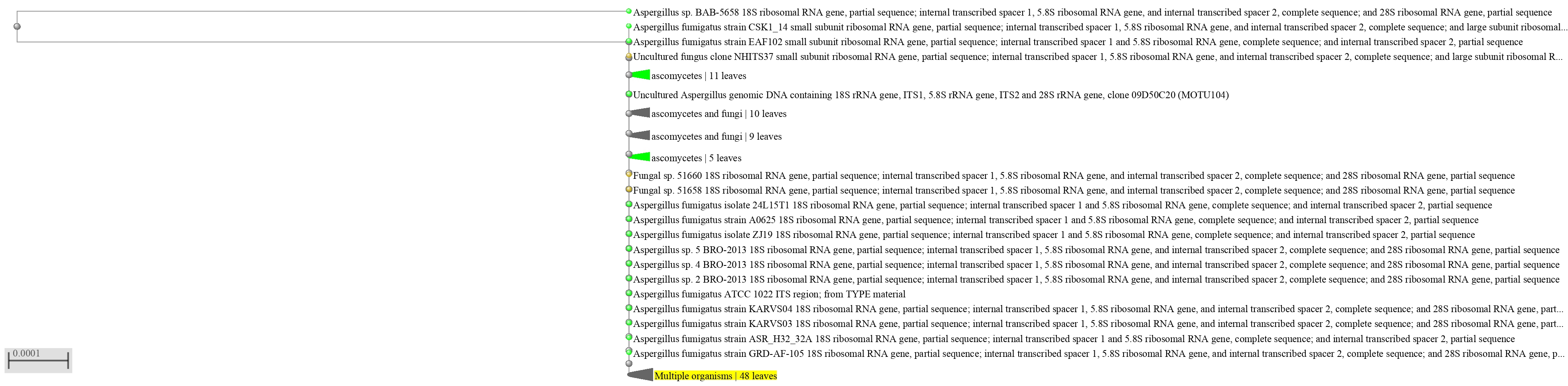

Supplement: Supplementary file 1 [file vetsci-07-00130-s001.zip › vetsci-905302-supplementary/Supplementary Figures/Figure 2.jpg]

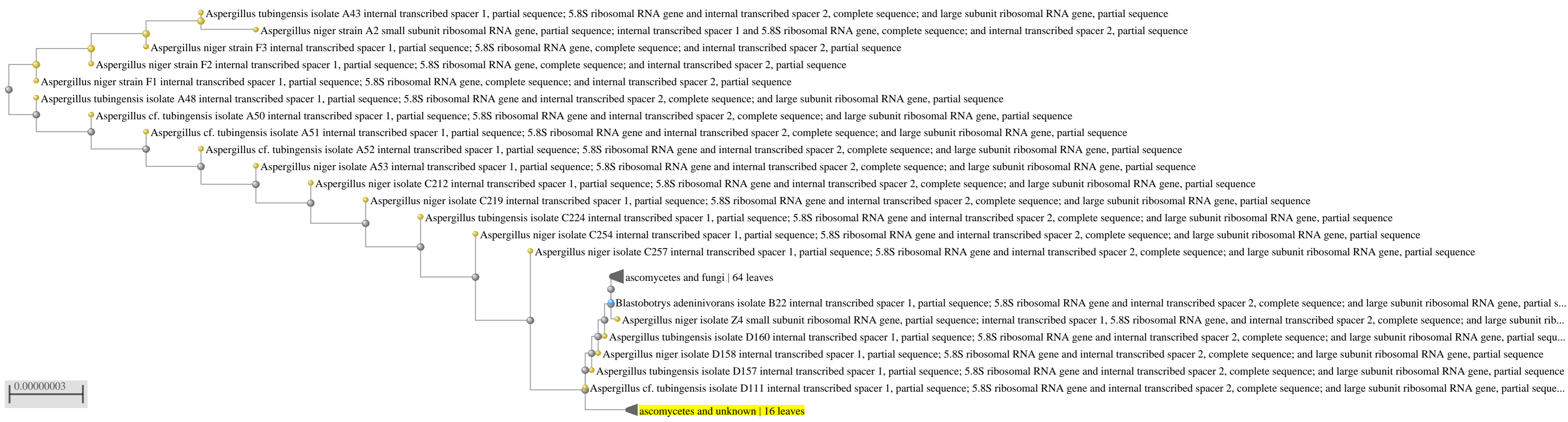

Supplement: Supplementary file 1 [file vetsci-07-00130-s001.zip › vetsci-905302-supplementary/Supplementary Figures/Figure 3 A.niger Tree.pdf]

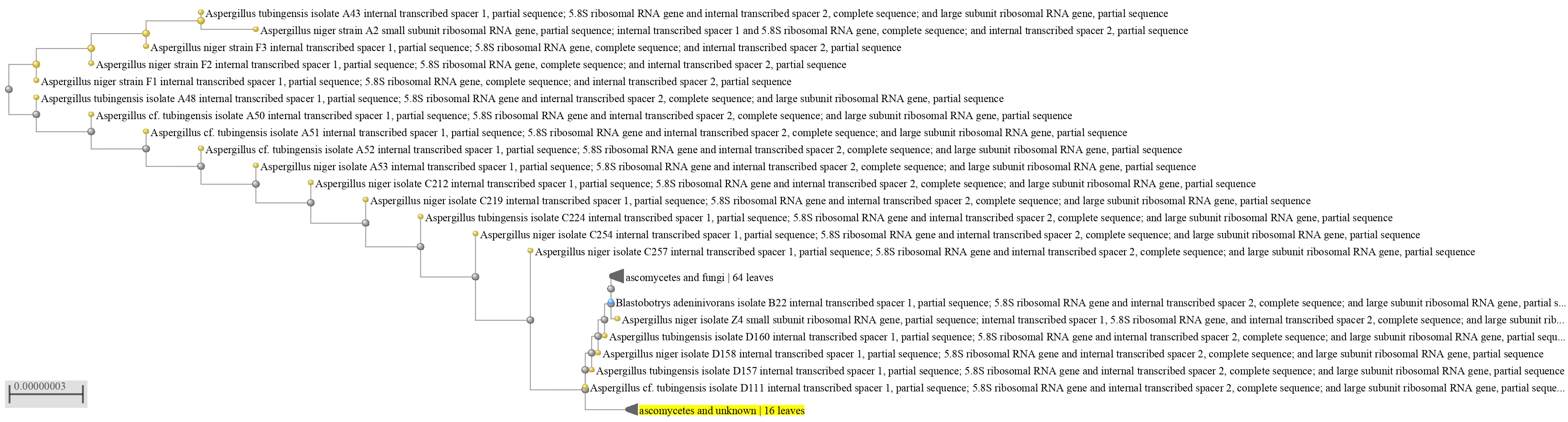

Supplement: Supplementary file 1 [file vetsci-07-00130-s001.zip › vetsci-905302-supplementary/Supplementary Figures/Figure 3.jpg]
